# Essential Oils from Pruning Residues of *Lavandula angustifolia* Mill. ‘Essence Purple’ and *Helichrysum italicum* (Roth) G.Don: Phytotoxic and Ecotoxicological Evaluation

**DOI:** 10.3390/molecules31081333

**Published:** 2026-04-18

**Authors:** Paola Malaspina, Flavio Polito, Annarita La Neve, Vincenzo De Feo, Laura Cornara, Domenico Trombetta, Antonella Smeriglio

**Affiliations:** 1 Department of Earth, Environment and Life Sciences, University of Genova, Corso Europa 26, 16132 Genova, Italy; paola.malaspina@unige.it (P.M.); laura.cornara@unige.it (L.C.); 2 Department of Pharmacy, University of Salerno, Via Giovanni Paolo II 132, 84084 Fisciano, Italy; defeo@unisa.it; 3 Department of Chemical, Biological, Pharmaceutical and Environmental Sciences (ChiBioFarAm), University of Messina, 98166 Messina, Italy; annarita.laneve@unime.it (A.L.N.); antonella.smeriglio@unime.it (A.S.)

**Keywords:** medicinal and aromatic plants, pruning residues, essential oils, glandular trichomes, phytochemical analysis, phytotoxic activity, *Artemia salina*, *Daphnia magna*, circular economy

## Abstract

Pruning residues from medicinal and aromatic plant cultivations represent an under-exploited biomass rich in bioactive metabolites. In this study, pruning by-products from *Lavandula angustifolia* Mill. ‘Essence Purple’ and *Helichrysum italicum* (Roth) G.Don were investigated as sources of essential oils (EOs) within a circular economy perspective. Micromorphological analyses confirmed the presence of secretory glandular trichomes in the residual biomass. EOs were obtained by steam distillation (0.33% and 0.15% yield for lavender and helichrysum, respectively) and chemically characterized by GC-FID and GC-MS. A total of 51 and 55 compounds were identified, accounting for 99.68% and 99.57% of the total composition. The main constituents were τ-cadinol (23.09%) and linalyl acetate (14.07%) in lavender EO and γ-curcumene (15.47%) and eudesm-4(14)-en-11-ol (10.71%) in helichrysum EO. Pruning-derived EOs showed a higher sesquiterpene content than those from conventional plant organs, indicating a compositional shift. Phytotoxic assays on *Hordeum vulgare*, *Raphanus sativus*, *Lolium multiflorum*, and *Sinapis alba* revealed concentration-dependent effects, with a stronger inhibition of radicle elongation than seed germination. These concentrations should be interpreted as indicative of intrinsic phytotoxic potential under controlled conditions. Ecotoxicological tests showed no significant reduction in viability in *Artemia salina*, whereas concentration- and time-dependent immobilization was observed in *Daphnia magna*, highlighting species-specific sensitivity, likely related to differences in the uptake and membrane interactions of lipophilic compounds. These findings highlight pruning residues as a promising biomass for the recovery of bioactive phytocomplexes with potential applications in sustainable weed management, although further studies under agronomically relevant conditions and comprehensive environmental assessments are required to validate their practical applicability.

## 1. Introduction

Italian floriculture represents a pillar of national agriculture, with a consolidated tradition that makes it one of the most important sectors. The regions of Northern Italy with the greatest overall importance in this field are Tuscany for nursery gardening—almost one billion euros in value (about 31% of the Italian market)—and Liguria for floriculture—almost 500 million euros (about 14.2%) [1]. In the Ligurian Region, the Western Agricultural and Horticultural District, established with the regional law 42/2001, identifies two main production hubs: the Sanremo area (Imperia province), specialized in the cultivation of flowers and cut foliage, and the Albenga area (Savona province), which is instead mainly focused on potted plants, with a particular specialization in aromatic herbs [1]. Indeed, since the 2000s, flower growers in the Albenga plain have progressively innovated the sector by introducing the cultivation of medicinal and aromatic plants (MAPs) in pots. As a result of this new production, significant quantities of plant by-products derived from pruning are obtained every year. In fact, the size and shape of potted plants must follow certain standards both to facilitate horticultural practices and to make them more suitable for transport to the Northern European market, which is one of the main export destination markets. Particularly Germany, followed by the Netherlands, France, and the UK, are leading importers of plant pots and a large consumer of fresh herbs [2].

The possibility of reusing and valorizing the pruning obtained from MAPs represents a new opportunity to be exploited from the perspective of the circular economy, according to the European Parliament Directives aimed to identify new strategies for a fair, healthy, and environmentally friendly food system [3]. In this context, a shift to a more sustainable approach to plant protection is desirable via Integrated Pest Management (IPM), a holistic approach to combat herbivores, pathogens, and weeds using sustainable methods, and reducing the use of synthetic pesticides [4].

By-products obtained by the pruning of cultivated MAPs, mainly consisting of their aerial parts, are in many cases important sources of essential oils (EOs), characterized by relatively low environmental persistence and a wide spectrum of biological activities, including phytotoxic, antibacterial, antifungal, and insecticidal activities, and, therefore, they can be useful tools for applications in organic farming [5].

For this purpose, our study aims to analyze the by-products derived from *Lavandula angustifolia* Mill. ‘Essence Purple’ (aerial parts) and *Helichrysum italicum* (Roth) G.Don (leaves), widely cultivated in the Albenga plain. The choice of these MAPs was also guided by the consideration that both plants (lavender and helichrysum) are included in the list of the most important MAPs in Italy in terms of Utilized Agricultural Area in 2019, according to AGEA data processing by Macaluso et al. [6].

Jiang et al. [7] summarizing the chemical characteristics and biological activities of lavender EO, highlighted its importance in different human health applications, but also reported the strong antifungal activity of this EO against *Fusarium solani* (Mart.) Sacc., which is the cause of Sorghum damping-off disease [8]. In addition, lavender EO can be used as a source of biological insecticides or for its repellent effect against mosquito and fly [9,10], while some data are also available on its phytotoxic activity on seeds of *Raphanus sativus*, *Lactuca sativa*, and *Lepidium sativum* [11].

As for helichrysum EO, it is known for its numerous antibacterial, antiviral, antifungal, and medicinal properties [12], which justify its wide use mainly in the cosmetics [13] and perfume industries [14]. Moreover, the insecticidal effects of helichrysum EO have also been reported [15]. Studies on its phytotoxic effects show that it is active against radicle elongation of radish [16] and that is able, at high concentrations, to cause seedling mortality of *Ailanthus altissima* [17], suggesting the importance to further investigating this field for the development of new plant-derived herbicides.

It should also be considered that natural EOs showed a remarkable phytochemical variability in relation to different factors, such as the cultivar, genetic and geographical origin, phenological stage, and even the growing conditions/season of plants [18,19]. Taking this into account, it becomes important to analyze the EOs obtained from different cultivars of the same species or from the same wild or cultivated species growing in areas characterized by different environmental conditions.

In the present study, the fragmented portions of lavender and helichrysum by-products from Albenga cultivations were first microscopically analyzed, and then their EOs were phytochemically characterized. The two EOs were subsequently evaluated for their phytotoxic effects on both weeds and crop species. In addition, a preliminary ecotoxicological assessment was performed using two aquatic invertebrate models (*Artemia salina* and *Daphnia magna*) to obtain complementary information on the environmental safety and biological activity of the investigated EOs. The final objective was to evaluate the possible use of these EOs as natural and environmentally friendly herbicides in organic farming practices.

## 2. Results

### 2.1. Micromorphological and Anatomical Investigations

#### 2.1.1. Main Features of *Lavandula angustifolia* Mill. ‘Essence Purple’

**Figure 1:**
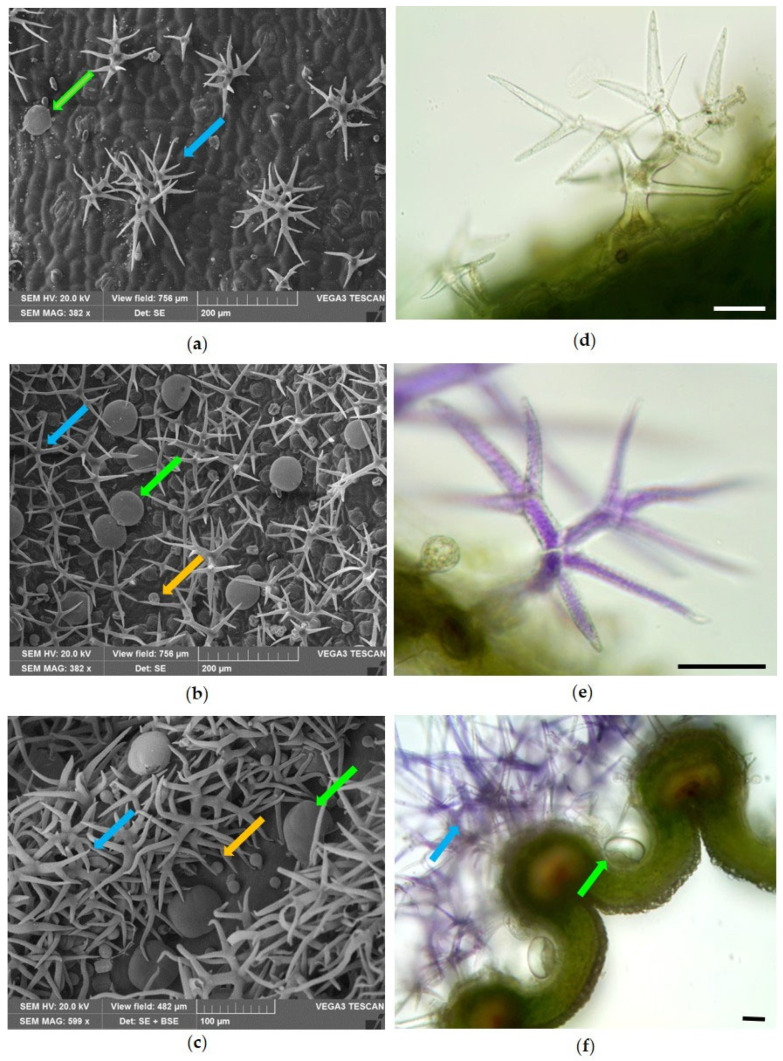
Scanning Electron Microscopy (**a**–**c**) and Light Microscopy (**d**–**f**) images of *Lavandula angustifolia* Mill. ‘Essence Purple’ showing the distribution of dendritic NGTs (blue arrows), PGTs (green arrows) and CGTs (yellow arrows) on (**a**) the adaxial surface of the leaf, (**b**) the abaxial surface of the leaf and (**c**) the external surface of the calyx. (**d**) Magnification of a dendritic NGT on the leaf surface; (**e**) magnification of a purple branched NGT on the calyx surface; (**f**) cross-section of the calyx showing a dense tomentum of purple dendritic NGTs overcoming the rib (blue arrow) and PGTs located between the ribs (green arrow). Bars = 50 µm.

All aerial portions of lavender were covered with non-glandular (NGTs) and glandular trichomes (GTs), both capitate (CGTs) and peltate (PGTs) (Figure 1, blue, yellow and green arrows, respectively). Analysis of the leaf surface revealed that the distribution of NGTs and GTs differed between the adaxial (Figure 1a) and abaxial surfaces (Figure 1b), with both types of trichomes being more abundant on the abaxial side. Furthermore, it was observed that, on the calyx, the NGTs formed a dense tomentum over the ribs; CGTs were uniformly distributed, while PGTs were more abundant between one rib and another (Figure 1c). The NGTs were multicellular and dendritic, with a central axis from which numerous arms arose, and a warty cuticle (Figure 1a–c, blue arrows). These trichomes were colorless on the leaf (Figure 1d), while they appeared purple on the external surface of the calyx (Figure 1e,f).

**Figure 2:**
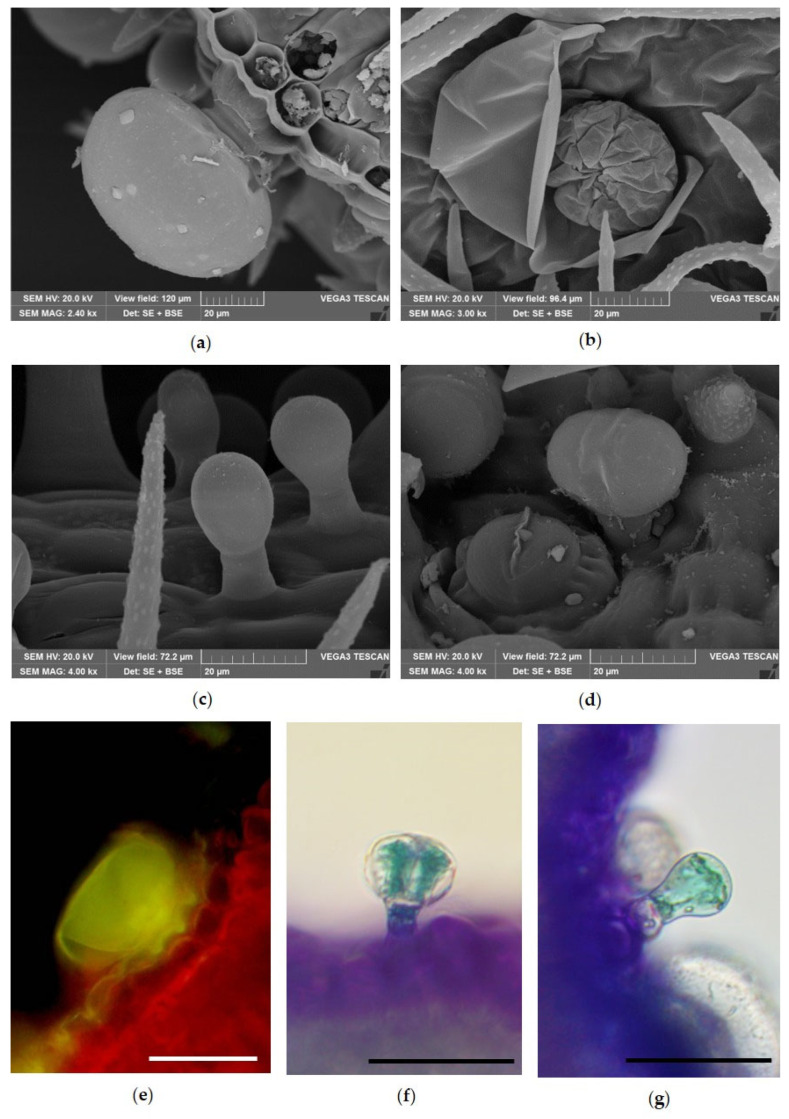
Scanning Electron Microscopy (**a**–**d**) and Light Microscopy (**e**–**g**) images showing the morphology and histochemistry of glandular trichomes on vegetative organs of *Lavandula angustifolia* Mill. ‘Essence Purple’. (**a**) Magnification of a PGT; (**b**) PGT with raised cuticle showing the eight secretory cells; (**c**) CGTs with spherical unicellular head; (**d**) CGT with a bicellular head; (**e**) PGT showing bright yellow fluorescence after staining with Fluorol Yellow; CGTs (**f**) with bicellular head and (**g**) with unicellular head stained blue-green with TBO. Bars = 50 µm (**e**–**g**).

Micromorphological and histochemical analyses showed the details of the different GTs detected on the leaf and calyx surface (Figure 2). In particular, peltate trichomes and two types of capitate trichomes were detected. PGTs were characterized by a basal cell, a short unicellular stalk and a head covered by a large subcuticular space in which EO accumulated (Figure 1a). After the cuticle ruptured and the secretion was released, eight secretory cells arranged in a radial pattern were well visible (Figure 2b). Two kinds of CGTs were found: type I consisted of a short unicellular stalk and a unicellular head cell with an oval to round shape (Figure 2c); type II consisted of a short unicellular stalk with a bicellular head (Figure 2d). Histochemical tests with Fluorol Yellow showed the presence of lipophilic substances within the glandular head of the PGTs (Figure 2e), while TBO highlighted the presence of phenolic compounds (Figure 2f,g) within the glandular heads of the CGTs.

**Figure 3:**
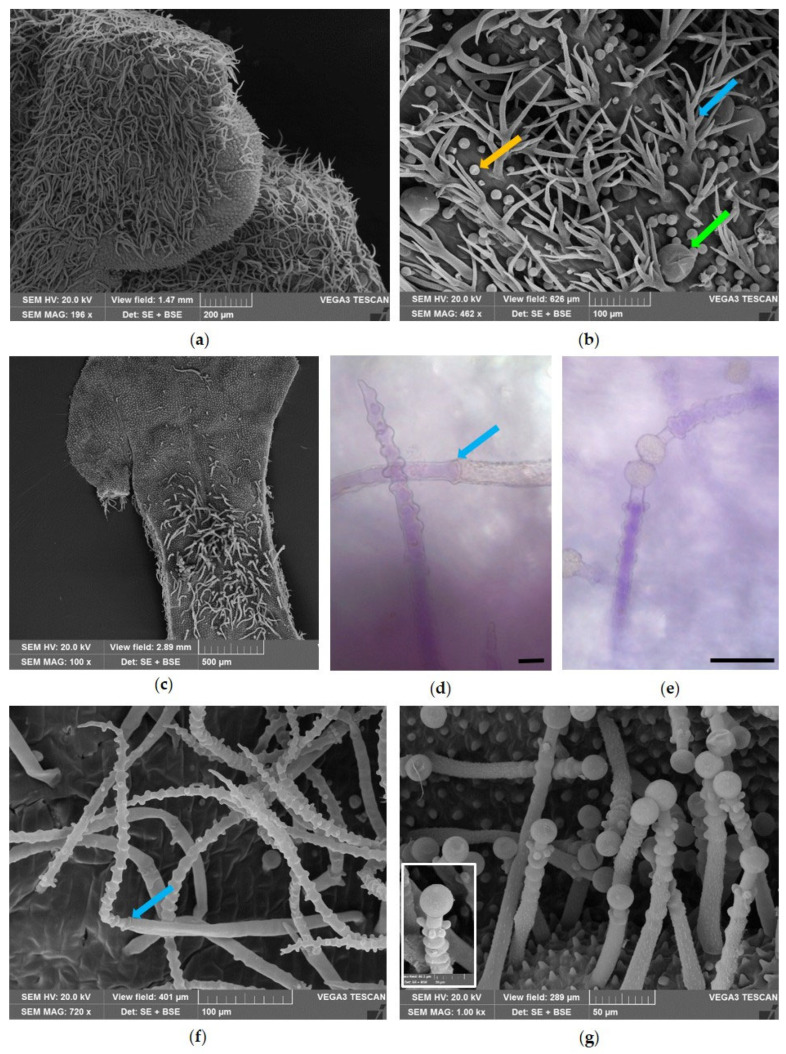
Scanning Electron Microscopy (**a**–**c**,**f**,**g**) and Light Microscopy (**d**,**e**) images of the corolla surface of *Lavandula angustifolia* Mill. ‘Essence Purple’. (**a**,**b**) External corolla surface showing a dense tomentum of dendritic NGTs (blue arrow) and the abundance of both PGTs (green arrow) and CGTs (yellow arrow). (**c**) General view of the internal corolla surface. (**d**) Magnification of long bicellular NGTs with a colorless warty basal cell and a purple apical cell, showing protruding knobs. Blue arrow indicates the ring of protruding knobs between the basal and apical cell. (**e**) Long-stalked CGTs with a smooth neck, a distinct unicellular head, and a ring of 4–6 protruding knobs between the stalk and the neck. (**f**) Several long bicellular NGTs; the blue arrow indicate the ring of protruding knobs which separate the long basal cell from the apical one. (**g**) Numerous long-stalked CGTs characterized by variability in the presence and density of protuberances, and in the frame, a magnification of the apical region of a glandular trichome. Bars = 50 µm (**d**,**e**).

The flower corolla showed a papillose surface with a different distribution and morphology of trichomes between the internal and external surface (Figure 3). The external surface showed a dense covering of NGTs and GTs (Figure 3a,b), similar to that described for the abaxial surface of the leaf, except for the NGTs, which were characterized by greater length and were appressed to the epidermis (Figure 3b). On the contrary, on the internal surface, the trichomes were mainly localized in the corolla tube (Figure 3c) and consisted of two different kinds (Figure 3d–g): a bicellular NGT (Figure 3d,f) and a multicellular CGT (Figure 3e,g). The NGTs showed a colorless basal cell, with a warty cuticle and a long apical cell stained purple by anthocyanins, and are characterized by small protuberances and a pointed apex. Between the basal and the apical cells of these trichomes there was a characteristic ring of protruding knobs (Figure 3d,f arrow). The CGTs consisted of a long warty stalk, showing more-or-less evident protuberances, a neck with variable length, and a unicellular glandular head. Between the stalk and the neck, there was a characteristic ring of from four to six protruding knobs. In addition, in these trichomes, anthocyanins were present only in the stalk cell (Figure 3e).

#### 2.1.2. Main Features of *Helichrysum italicum* (Roth) G.Don

**Figure 4:**
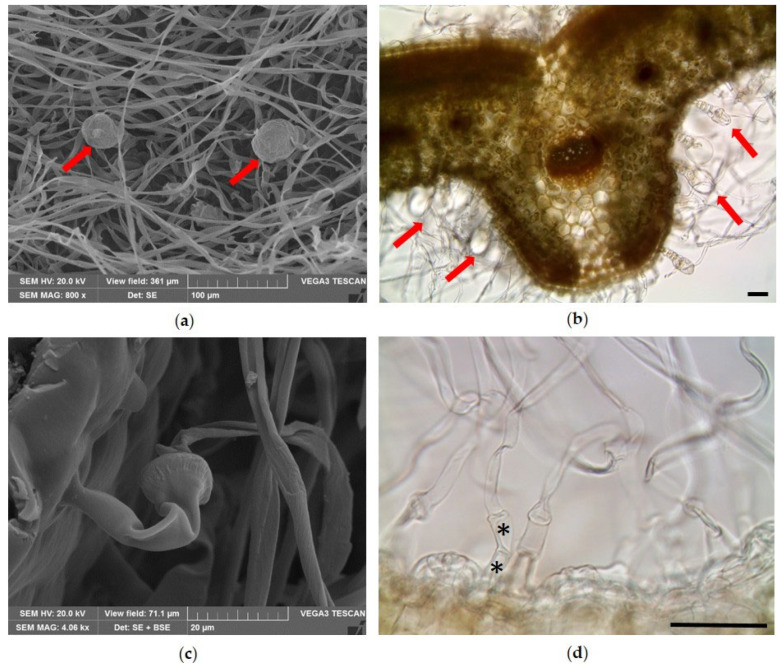
Scanning Electron Microscopy (**a**,**c**) and Light Microscopy (**b**,**d**) images of the leaf of *Helichrysum italicum* (Roth) G.Don. (**a**) Abaxial surface covered with a dense tomentum of filamentous NGTs and some BGTs (red arrows). (**b**) Transverse section showing numerous BGTs on the lower epidermis (red arrows). (**c**) Detail of an NGT with the basal portion spindle-shaped. (**d**) NGTs highlighting the presence of two basal cells (asterisks). Bars = 50 µm (**b**,**d**).

The leaf surface of helichrysum was covered by a dense wooly tomentose indumentum composed of very long and filamentous multicellular uniseriate NGTs (Figure 4a). Scattered and partially hidden among the covering of NGTs, the glandular trichomes were also identified (Figure 4a, red arrows). The leaf showed revolute margins defining two crypts within which numerous glandular trichomes were visible among the tomentum of the NGTs (Figure 4b, red arrows). The NGTs showed a bicellular basal portion, spindle-shaped, and an apical portion made of an elongated apical cell, slightly twisted (Figure 4c,d).

**Figure 5:**
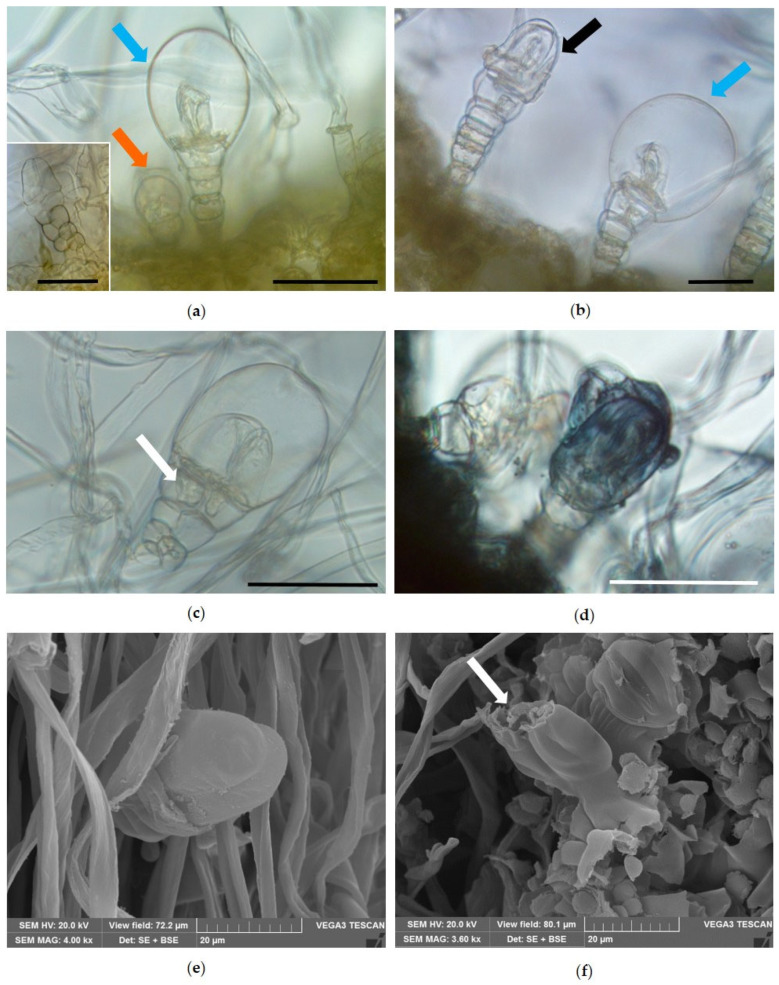
Light microscopy (**a**–**d**) and Scanning Electron Microscopy (**e**,**f**) images of BGTs on the leaves of *Helichrysum italicum* (Roth) G.Don. (**a**) BGTs type I and type II (blue and orange arrows, respectively); in the frame, the biseriate structure of type I BGTs is visible. (**b**) BGTs type I with cuticle attached to the head cells (black arrow), and another one with a wide subcuticular space where the secretion accumulates (blue arrow). (**c**) Magnification of a BGT with traces of secreted material in the cells of the middle zone (white arrow). (**d**) The secretion of type I BGT reacts positively with Sudan Black. (**e**) Magnification of the apical head secretory cells. (**f**) A type I BGT in which an apical rupture in the cuticle is visible (white arrow). Bars = 50 µm (**a**–**d**).

Two types of biseriate glandular trichomes (BGTs) were observed (Figure 5): type I (Figure 5a, blue arrow and frame) showed a basal zone with a single stalk of 1 or 2 cells, followed by a median zone of variable length, with four or five parallel pairs of cells, culminating in a multicellular head with a pair of secretory cells. The apical pair of these cells, longer than the others, developed a large subcuticular space, where secretion accumulated (Figure 5a, blue arrow). BGTs type II (Figure 5a, orange arrow) were found only occasionally; they were smaller in size and showed a narrower subcuticular space than type I.

Regarding BGTs type I, fully developed glandular trichomes were observed in which the cuticle appeared to adhere to the apical secretory cells (Figure 5b, black arrow). On the other hand, other BGTs type I appeared club-shaped due to the detachment of the cuticle from the outer wall of the two head cells, which resulted in a subcuticular space in which the secretion accumulated (Figure 5b, blue arrow). The presence of traces of secreted material in the cells of the middle zone of BGTs type I (Figure 5c, white arrow) suggests that these cells participate in the secretory process and that the secretory products are transferred to the apical cells through plasmodesmata. In the apical cells, the secretion finally accumulates in the subcuticular space and reacts positively with Sudan Black, confirming its lipophilic nature (Figure 5d). Finally, SEM observations highlighted the morphology of the apical head cells, which were longer than the others (Figure 5e), and revealed the presence of an apical rupture in the cuticle from which the secretion is released (Figure 5f, arrow).

### 2.2. Phytochemical Analyses

The chemical compositions of EOs from *L. angustifolia* ‘Essence Purple’ and *H. italicum* are reported in Table 1 and Table 2, respectively.

In lavender EO, obtained with a yield of 0.33%, 51 compounds were identified by GC–MS and quantified by GC-FID, representing 99.68% of the total composition.

The main classes of compounds were oxygenated monoterpenes (35.36%), oxygenated sesquiterpenes (28.47%), and hydrocarbon sesquiterpenes (19.70%). The major compound was τ-cadinol (23.09%), followed by linalyl acetate (14.07%), γ-cadinene (12.07%), and linalool (7.37%). All the remaining compounds were present in percentages lower than 5%.

In the EO obtained from helichrysum, a total of 55 compounds were identified by GC–MS and quantified by GC–FID, representing 99.57% of the total EO composition.

The most represented class was hydrocarbon sesquiterpenes (42.86%), followed by oxygenated sesquiterpenes (35.04%) and oxygenated monoterpenes (11.30%). The major compound was γ-curcumene (15.47%), followed by eudesm-4(14)-en-11-ol (10.71%). Other compounds present in percentages higher than 5% were italicene (8.42%), neryl acetate (8.33%), rosifoliol (7.37%), and guaiol (5.83%).

### 2.3. Phytotoxicity

**Figure 6:**
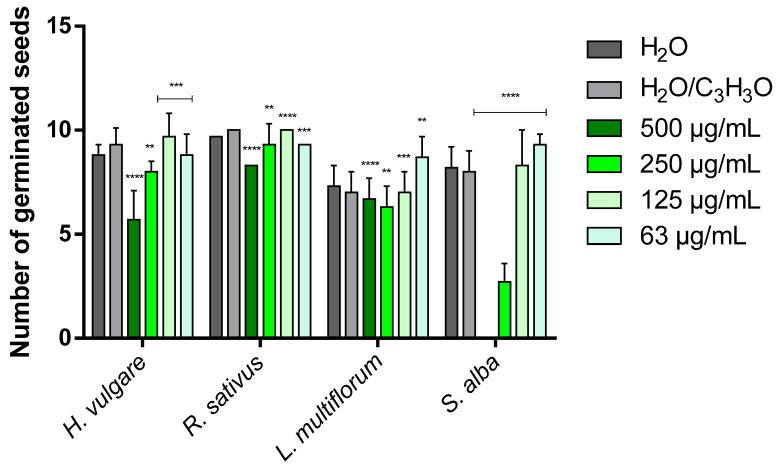
Bar graph showing the phytotoxic activity of *Lavandula angustifolia* Mill. ‘Essence Purple’ EO on the seed germination of *H. vulgare*, *R. sativus*, *L. multiflorum* and *S. alba*. Data are expressed as mean ± standard deviation (SD) of three independent experiments. ** *p* < 0.01; *** *p* < 0.001; **** *p* < 0.00001 vs. control (ANOVA followed by Dunnett’s multiple comparison test).

**Figure 7:**
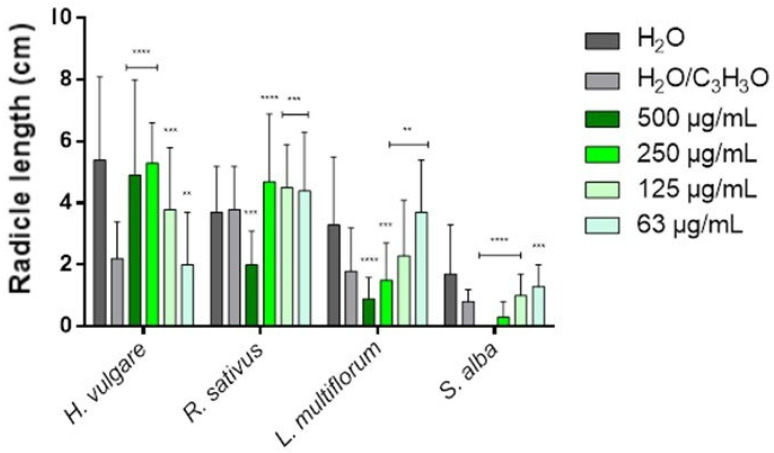
Bar graph showing the phytotoxic activity of *Lavandula angustifolia* Mill. ‘Essence Purple’ EO on the radicle elongation of *H. vulgare*, *R. sativus*, *L. multiflorum* and *S. alba*. Data are expressed as mean ± standard deviation (SD) of three independent experiments. ** *p* < 0.01; *** *p* < 0.001; **** *p* < 0.00001 vs. control (ANOVA followed by Dunnett’s multiple comparison test).

**Figure 8:**
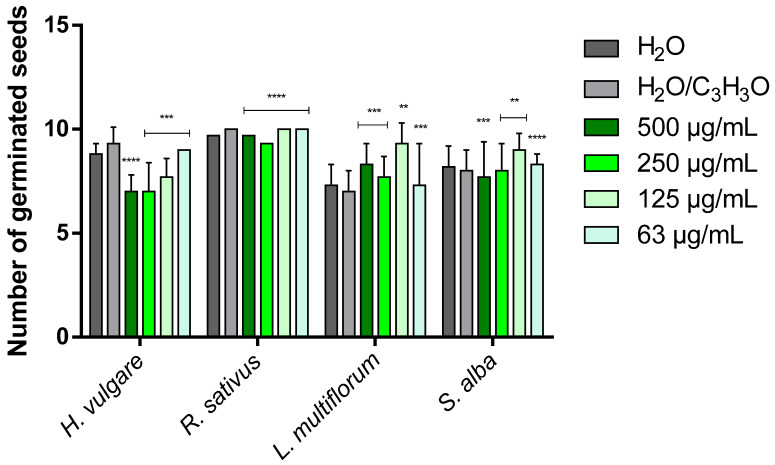
Bar graph showing the phytotoxic activity of *Helichrysum italicum* (Roth) G.Don EO on the seed germination of *H. vulgare*, *R. sativus*, *L. multiflorum* and *S. alba*. Data are expressed as mean ± standard deviation (SD) of three independent experiments. ** *p* < 0.01; *** *p* < 0.001; **** *p* < 0.00001 vs. control (ANOVA followed by Dunnett’s multiple comparison test).

**Figure 9:**
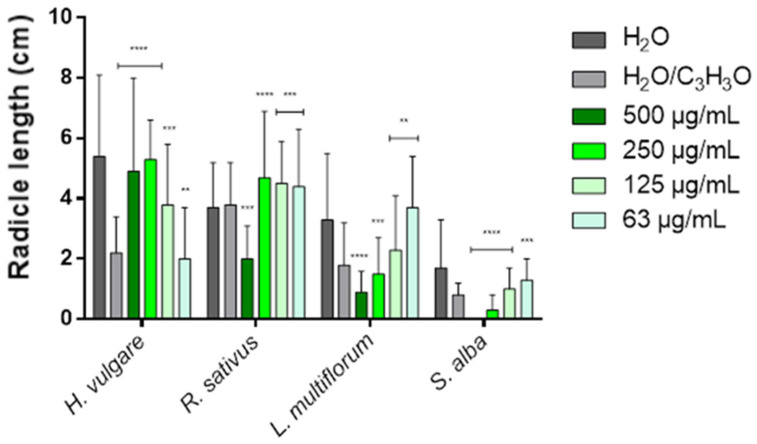
Bar graph showing the phytotoxic activity of *Helichrysum italicum* (Roth) G.Don EO on the radicle elongation of *H. vulgare*, *R. sativus*, *L. multiflorum* and *S. alba*. Data are expressed as mean ± standard deviation (SD) of three independent experiments. ** *p* < 0.01; *** *p* < 0.001; **** *p* < 0.00001 vs. control (ANOVA followed by Dunnett’s multiple comparison test).

Table 3 and Table 4 show the phytotoxic activity of EOs on the germination and radicle elongation processes of the four selected seeds: the two crops *Hordeum vulgare* L. and *Raphanus sativus* L. and the two weeds *Lolium multiflorum* Lam. and *Sinapis alba* L. In the tables, the results are reported as percentage inhibition (%) relative to the treatment carried out with the control solution consisting of water and acetone (99.5:0.5 *v*/*v*), to which an inhibition value of 0.0% was assigned. To simplify the visualization of the data, heat maps were generated for each table using green to indicate positive inhibition values and red to indicate negative inhibition values (i.e., stimulation of the process). White indicates the absence of activity (0.0%). The more intense the color, the greater the activity. Figure 6, Figure 7, Figure 8 and Figure 9 instead represent the same effects of the EO solutions on the germination and radicle elongation processes of the seeds using bar graphs constructed from the direct measurements obtained from the seeds (number of germinated seeds and radicle length expressed in cm).

*Lavandula angustifolia* ‘Essence Purple’ EO showed a concentration-dependent phytotoxic effect.

In general, at low concentrations (125 and 63 µg/mL), limited inhibitory effects or stimulation phenomena were observed, whereas inhibitory effects predominated at higher concentrations (500 and 250 µg/mL). However, the response varied depending on the plant species. The germination of *H. vulgare* was only slightly affected at the lowest concentrations (125 and 63 µg/mL), while the two highest concentrations (500 and 250 µg/mL) showed a moderate inhibitory effect (38.7% and 14.0%, respectively). In contrast, radicle elongation was slightly inhibited (9.1%) at the lowest concentration (63 µg/mL) and strongly stimulated at the other concentrations (72.7–140.9%).

The germination of *R. sativus* was also weakly influenced by the EO, with inhibition values not exceeding 17.0%. Conversely, radicle elongation was stimulated at all concentrations (from −15.8% to −23.7%) and was inhibited by almost half (47.4%) only at 500 µg/mL. In *L. multiflorum*, both germination and radicle elongation were stimulated at 125 and 63 µg/mL, with a wide range of stimulation (from −24.3% to −105.6%). At higher concentrations (500 and 250 µg/mL), inhibitory effects were observed, reaching up to 50.0% inhibition in the case of radicle elongation. *S. alba* was the most sensitive species to the phytotoxic action of the EO. While low concentrations (125 and 63 µg/mL) stimulated germination and radicle elongation (from −3.8% to −62.5%), concentrations of 500 and 250 µg/mL strongly inhibited both processes, with inhibition values ranging from 62.5% up to complete inhibition (100%).

The EO from *H. italicum* also showed concentration-dependent effects, with stimulation phenomena prevailing at low concentrations (125 and 63 µg/mL), particularly on radicle elongation, and inhibitory effects appearing at higher concentrations (500 and 250 µg/mL). Overall, its phytotoxic activity was lower than that observed for lavender EO. *H. vulgare* was the species most sensitive to the action of *H. italicum* EO, showing inhibition of germination at all tested concentrations, with a maximum inhibition of 24.7%. Conversely, radicle elongation was strongly stimulated at all concentrations, reaching a maximum stimulatory effect of −131.8%. The germination of *R. sativus* was only slightly affected, with a modest inhibition (7.0%) observed at the highest concentration (500 µg/mL). Radicle elongation, on the other hand, was stimulated at the lowest concentrations (125 and 63 µg/mL), with stimulation values of −31.6% and −26.3%, respectively, while the highest concentration produced the strongest inhibitory effect (25.0%).

The germination of *L. multiflorum* was stimulated at all tested concentrations, with stimulation values ranging from −4.3% to −32.8%. Radicle elongation was also strongly stimulated at the lowest concentrations (up to −122.2%), whereas at 500 µg/mL, a clear inhibitory effect was observed (27.8%). Finally, in *S. alba*, the lowest concentrations (125 and 63 µg/mL) stimulated both germination and radicle elongation (from −3.8% to −62.5%). At the highest concentration, however, an inhibitory effect was observed: mild for germination (3.8%), but more pronounced for radicle elongation (25.0%).

Overall, *L. angustifolia* ‘Essence Purple’ EO exhibited the strongest inhibitory activity, affecting both germination and radicle growth processes, particularly in weed species, with *S. alba* being the most sensitive. In contrast, the EO from *H. italicum* showed more limited inhibitory effects, mainly observable at the highest concentrations and primarily affecting radicle elongation (except in *H. vulgare*).

These results highlight two main aspects. First, the cultivated species (*H. vulgare* and *R. sativus*) did not show strong inhibitory responses, but rather several stimulation phenomena, suggesting that, at the tested concentrations, the EOs do not exert marked phytotoxic effects on these crops and may even promote early seedling development. Second, the most pronounced inhibitory effects were observed in weed species, particularly *S. alba*, indicating a potential selective phytotoxic activity against weeds. These findings suggest that, at concentrations higher than those tested in the present study, a stronger phytotoxic activity may occur, making these EOs potential candidates for weed control applications. Further studies are therefore required to confirm these observations and to evaluate their activity at higher concentrations under conditions closer to practical applications.

### 2.4. Ecotoxicological Effects

The ecotoxicological effects of the investigated EOs were assessed using two aquatic invertebrate models, *Artemia salina* and *Daphnia magna*.

**Figure 10:**
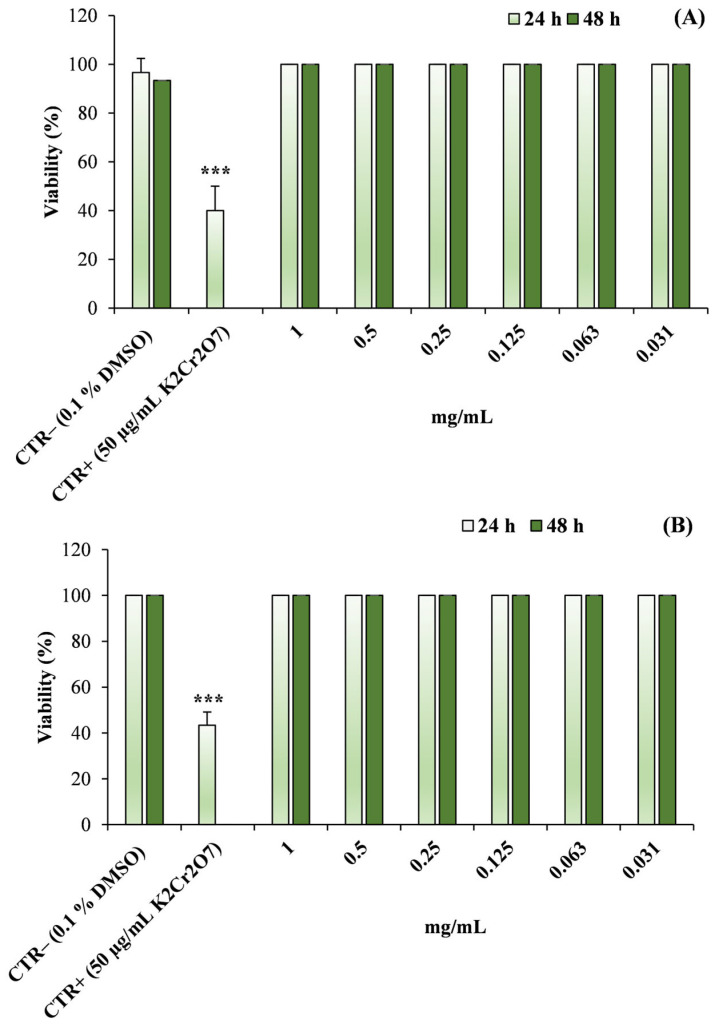
Viability of *Artemia salina* nauplii after exposure to different concentrations of (**A**) *Lavandula angustifolia* Mill. ‘Essence Purple’ and (**B**) *Helichrysum italicum* (Roth) G.Don essential oils (EOs) for 24 and 48 h. Data are expressed as mean ± standard deviation (SD) of three independent experiments performed in triplicate. DMSO (0.1%) was used as negative control (CTR−), while potassium dichromate (K_2_Cr_2_O_7_, 50 µg/mL) was used as positive control (CTR+). Statistical significance refers to the comparison with negative control (*** *p* < 0.001).

Exposure of *A. salina* nauplii to increasing concentrations of *L. angustifolia* ‘Essence Purple’ and *H. italicum* EOs (0.03125–1 mg/mL) did not result in any detectable reduction in organism viability after either 24 or 48 h of incubation (Figure 10).

Under all tested conditions, the percentage of viable nauplii remained comparable to that observed in the negative control (0.1% DMSO), and no statistically significant differences were detected among treatments. In contrast, the positive control (K_2_Cr_2_O_7_, 50 µg/mL) produced the expected toxic response, resulting in a statistically significant decrease in nauplii viability compared with the negative control (*p* < 0.001), thus confirming the reliability of the assay.

**Figure 11:**
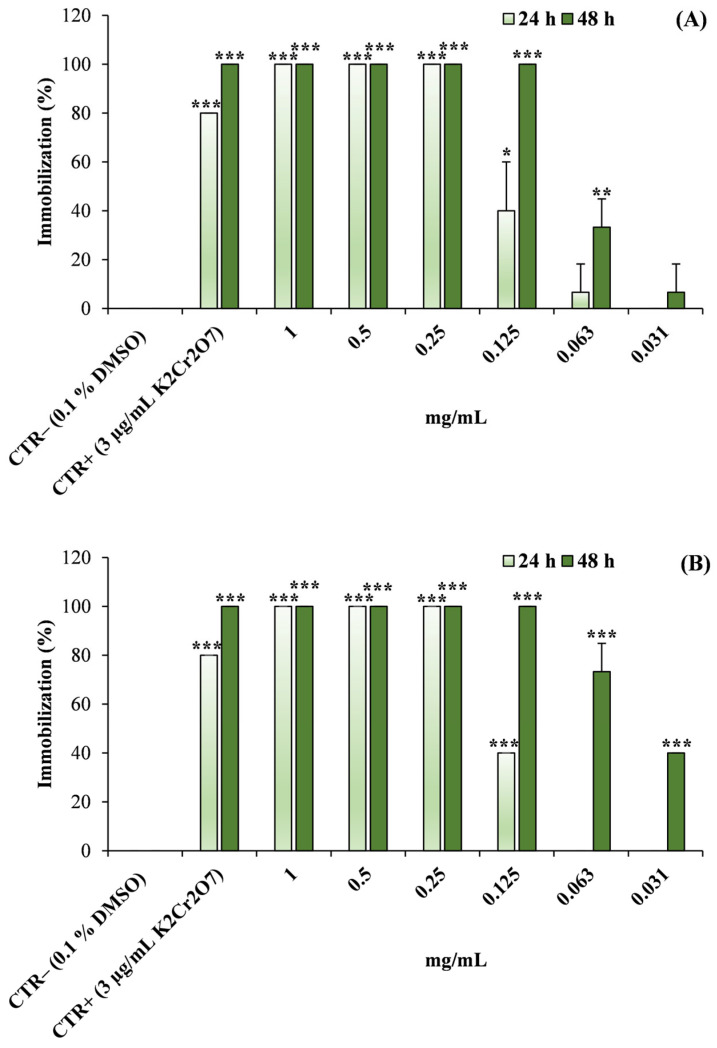
Immobilization of *Daphnia magna* neonates after exposure to different concentrations of (**A**) *Lavandula angustifolia* Mill. ‘Essence Purple’ and (**B**) *Helichrysum italicum* (Roth) G.Don essential oils (EOs) for 24 and 48 h. Data are expressed as mean ± standard deviation (SD) of three independent experiments performed in quadruplicate. DMSO (0.1%) was used as negative control (CTR−), while potassium dichromate (K_2_Cr_2_O_7_, 3 µg/mL) was used as positive control (CTR+). * *p* < 0.05; ** *p* < 0.01; *** *p* < 0.001 vs. negative control (CTR−).

A different response pattern was observed in the *Daphnia magna* assay (Figure 11).

Exposure to both EOs resulted in a significant increase in organism immobilization compared with the negative control. For all tested concentrations and time points, the percentage of immobilized individuals was statistically significantly higher than that observed in the control (*p* < 0.05–*p* < 0.001). Moreover, the magnitude of the effect increased with concentration and became more pronounced after 48 h of exposure, indicating a clear concentration- and time-dependent response. At the highest concentrations tested, high levels of immobilization were observed, while the positive control (K_2_Cr_2_O_7_, 3 µg/mL) produced the expected strong toxic effect.

The median effective concentration (EC_50_) values calculated from the concentration–response curves for *D. magna* were 0.135 mg/mL (95% CI: 0.124–0.146 mg/mL) and 0.064 mg/mL (95% CI: 0.057–0.072 mg/mL) for *L. angustifolia* ‘Essence Purple’ EO after 24 and 48 h of exposure, respectively, and 0.147 mg/mL (95% CI: 0.137–0.158 mg/mL) and 0.039 mg/mL (95% CI: 0.035–0.044 mg/mL) for *H. italicum* EO at the same time points.

## 3. Discussion

Significant quantities of plant by-products are generated every year from the cultivation of MAPs in pots. Such materials often remain rich in bioactive compounds that can be exploited for a wide range of applications [20,21]. In many horticultural production systems, however, pruning residues are still treated as low-value waste or are simply discarded, even though these materials retain a substantial fraction of the specialized metabolites originally synthesized by the plant. The valorization of such residues therefore represents an important opportunity both from an environmental perspective and for the recovery of valuable natural compounds. In this context, pruning-derived biomass may also represent a source of chemically differentiated EOs compared to conventional plant matrices, thus expanding the spectrum of exploitable natural products.

Pharmacognostic investigations of the pruning residues obtained from lavender and helichrysum confirmed that these materials can still represent a suitable source for EO extraction by hydrodistillation. Micromorphological analyses revealed the presence of numerous GTs in the aerial portions of both species, indicating that the structures responsible for the synthesis and accumulation of volatile metabolites remain abundant even in secondary biomass derived from pruning. In aromatic plants, GTs play multiple ecological and physiological roles, such as plant protection and allopathic interactions with neighboring plants [22,23,24]. These findings collectively confirm that pruning residues retain a fully functional secretory apparatus, supporting their suitability as alternative raw materials for EO extraction.

In all aerial portions of lavender analyzed in this study, characteristic dendritic NGTs and different types of GTs were observed, in agreement with previous descriptions reported by Giuliani et al. [25,26] and Blazekovic et al. [27]. In addition, similarly to what was reported by Rahfeld [28] and Blazekovic et al. [27], another type of CGT was detected on the internal surface of the corolla tube. Conversely, the CGT described by Blazekovic et al. [27] for *Lavandula × intermedia* ‘Budrovka’ was not observed in *L. angustifolia* ‘Essence Purple’. Histochemical tests further confirmed that PGTs represent the main sites of EO production, whereas CGTs appear to be richer in phenolic compounds.

In the leaves of *Helichrysum italicum*, the same two types of BGTs described by Rodrigues et al. [29] were found, with type I BGTs particularly abundant within the crypts formed by the revolute leaf margins, whereas type II BGTs were present only occasionally. Type I BGTs were consistent with those described by Perrini et al. [30] for *H. italicum* ssp. *microphyllum*. Comparable structures were reported for *H. stoechas* by Ascensão et al. [31] and subsequently by Rodrigues et al. [29] for *H. italicum* ssp. *picardii*. Histochemical staining confirmed the lipophilic nature of the secretions accumulated in these trichomes, supporting their key role in EO biosynthesis.

The NGTs observed in *H. italicum* were similar to those reported for related species such as *H. aureonitens* and *H. splendidum* [32,33]. As also noted by Mashigo et al. [33], these structures showed a characteristic spindle-shaped basal portion. The dense indumentum covering the leaf surface of *H. italicum* likely plays an important adaptive role, protecting the leaves against excessive water loss and intense solar radiation, two major environmental stressors in Mediterranean habitats [31,34].

The valorization of agro-industrial by-products is increasingly important within the circular economy, which promotes the recovery of valuable resources from materials traditionally treated as waste [35,36]. MAPs can be promising sources of secondary biomass potentially rich in specialized metabolites with biological activity [37]. In the case of lavender and helichrysum, most studies have focused on the recovery of phenolic and other non-volatile compounds from distillation waste and their biological or bioenergetic applications [38,39,40,41,42,43,44], while the volatile fraction derived from pruning residues remains poorly studied.

A direct comparison with EOs obtained from conventional plant organs is therefore necessary to fully contextualize the added value of pruning-derived materials.

*L. angustifolia* EOs are most often extracted from the flowering tops, and several studies demonstrate that these EOs, despite their genotype-dependent, growing area-dependent, and climatic conditions [45], are generally characterized by a clear predominance of oxygenated monoterpenes [46]. Recent analyses report that EOs obtained from flowering tops are dominated by monoterpenes, particularly linalyl acetate and linalool, which together can represent over 70–80% of the total composition, while sesquiterpene fractions are relatively low (~2–3%) [47,48,49,50]. In addition, ornamental cultivars may display significant chemotypic variability [51], while seasonal factors and harvest timing can further influence EO composition [52]. In the present study, EO obtained from *L. angustifolia* pruning residues showed a partially different profile compared to the ones typical of EOs obtained from flowering tops. Although linalool and linalyl acetate were still present, a relatively higher contribution of sesquiterpenes was observed, suggesting a compositional shift associated with the use of secondary biomass.

A similar trend was observed for helichrysum. EOs obtained from its flowering tops or aerial parts are generally characterized by monoterpenes such as neryl acetate, nerol, geraniol, nerolidole, and α-pinene, along with sesquiterpenes such as γ-curcumene, β-caryophylene, and β-selinene, with a qualitative and quantitative composition varying depending on the chemotype and environmental conditions [12,53,54,55]. The EO obtained from pruning residues in this study, while reflecting a profile consistent with some chemotypes in the literature, showed a predominance of sesquiterpenes, particularly γ-curcumene.

These variations in composition can be explained by the nature of the distilled material, which included not only inflorescences, but also leaves and partially lignified stems—tissues known to contain higher proportions of sesquiterpenes than the floral parts alone [56].

Overall, pruning residues appear to generate EOs with distinct chemical profiles, potentially associated with differentiated biological activities compared to those obtained from conventional plant organs. Studies on the phytotoxic activity of EOs obtained from lavender and helichrysum pruning residues are lacking, as most of the available literature concerns EOs derived from conventional plant organs or non-volatile fractions derived from plant residues [57]. The EO of *L. angustifolia* has previously shown inhibitory effects on the germination of several plant species, including crops such as *H. vulgare* and *Triticum aestivum* [58], as well as *R. sativus* and *Lactuca sativa* [11], and weeds such as *L. multiflorum* [59]. These effects have often been associated with EOs rich in oxygenated monoterpenes such as linalool and linalyl acetate [60]. The EOs analyzed in this study displayed a relevant contribution of sesquiterpenes in addition to oxygenated monoterpenes. Due to their higher lipophilicity and lower volatility, sesquiterpenes can persist longer in the substrate and are often more active on post-germinative processes, such as radicle elongation, rather than on seed germination [60]. Consequently, the inhibitory effects observed in the present tests were generally more pronounced on root elongation than on germination.

The concentrations tested in this study (63–500 µg/mL), although consistent with laboratory screening approaches, should be interpreted as being indicative of intrinsic phytotoxic potential rather than directly transferable to agronomic conditions. In field scenarios, environmental dispersion, soil interactions, volatility, and formulation factors are expected to significantly influence the effective concentrations at the target site. Therefore, further studies aimed at optimizing formulation strategies and validating efficacy under greenhouse and field conditions are required.

Interestingly, several treatments at lower concentrations resulted in stimulatory effects on germination or radicle elongation. Such responses are frequently described in allopathic studies and may be interpreted as hormetic effects, a biphasic concentration–response phenomenon in which low concentrations of bioactive compounds stimulate biological processes whereas higher concentrations exert inhibitory effects [61,62].

Available studies on the phytotoxic activity of *H. italicum* EO are relatively limited. Mancini et al. [16] reported modest inhibitory effects on radicle elongation in *R. sativus* and *Lepidium sativum*, whereas Karalija et al. [17] observed inhibition of post-germinative growth in *Ailanthus altissima*. The relatively moderate phytotoxic activity detected in the present work is therefore consistent with the literature.

In addition to phytotoxicity, the present study also included a preliminary ecotoxicological evaluation using *Artemia salina* and *Daphnia magna*, which are widely employed as screening organisms for evaluating the biological activity and environmental safety of natural products [63,64,65]. The two models displayed different sensitivity profiles. In the *A. salina* assay, neither EO produced significant reductions in nauplii viability within the tested concentration range. Conversely, a clearer response was observed in *Daphnia magna*, where both EOs induced concentration-dependent immobilization.

The different sensitivity observed between *A. salina* and *D. magna* may be explained by both physiological and ecological differences between marine and freshwater crustaceans, as well as by species-specific variations in membrane composition, permeability, and detoxification capacity. In particular, *D. magna* is characterized by a high filtration rate and a thin integument, which may facilitate the uptake of lipophilic compounds such as terpenoids, thereby increasing its sensitivity. In contrast, *A. salina* exhibits greater tolerance to environmental stressors, including xenobiotics, which may partially account for its lower sensitivity under the tested conditions.

The EC_50_ values obtained for *D. magna* decreased between 24 and 48 h of exposure, indicating a time-dependent toxic response. Differences in sensitivity between aquatic invertebrate species have been widely reported in ecotoxicological studies [64,66]. From a mechanistic perspective, the lipophilic nature of EO constituents, particularly sesquiterpenes, may promote their interaction with biological membranes, potentially altering membrane fluidity, permeability, and cellular homeostasis. This mechanism may contribute to the observed immobilization effects in *D. magna* [67,68].

From an environmental perspective, these results should be interpreted within the framework of a preliminary screening of biologically active natural mixtures. EOs are characterized by relatively rapid environmental degradation compared with many synthetic pesticides [69,70].

However, their natural origin does not necessarily imply ecological safety, and the observed effects on *D. magna* highlight the need for more comprehensive ecotoxicological assessments, including chronic exposure studies and evaluation under environmentally relevant conditions, before any potential agronomic application.

Overall, the results demonstrate that pruning residues from ornamental cultivations of *L. angustifolia* ‘Essence Purple’ and *H. italicum* represent a valuable secondary biomass that can be exploited for the recovery of bioactive EOs.

While the present findings provide a solid proof-of-concept, further investigations are required to bridge the gap between laboratory evidence and practical applications.

In a broader perspective, the valorization of pruning residues from aromatic plants may contribute to the development of sustainable strategies aligned with circular economy principles, transforming horticultural waste into sources of natural compounds with potential agronomic applications.

## 4. Materials and Methods

### 4.1. Plant Material

**Figure 12:**
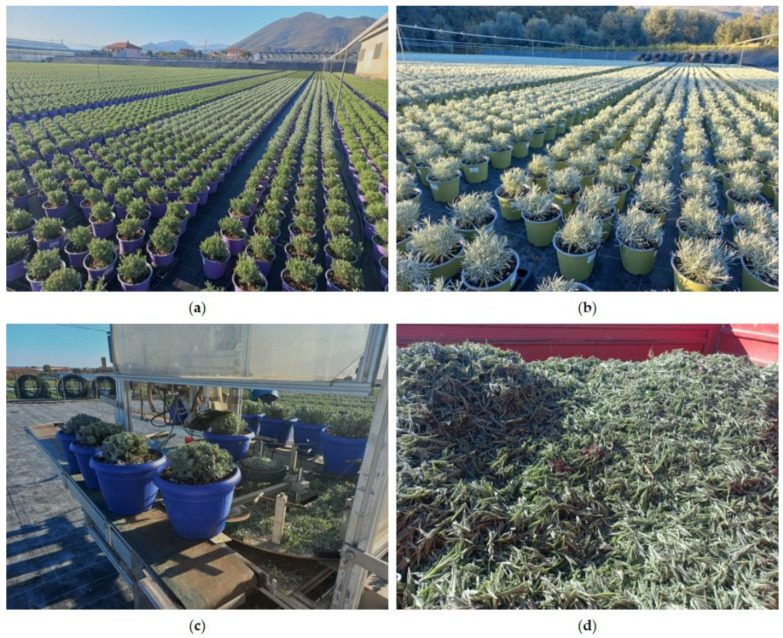
Cultivation and pruning residues of the investigated aromatic plants. (**a**) Cultivation of *Lavandula angustifolia* Mill. ‘Essence Purple’ in pots in the Albenga plain (Liguria, Italy). (**b**) Cultivation of *Helichrysum italicum* (Roth) G.Don in pots in the same production area. (**c**) Mechanical pruning of potted plants to maintain the commercial rounded shape. (**d**) Pruning residues collected from cultivation and used as plant material for essential oil extraction.

The pruning material derived from *Lavandula angustifolia* Mill. ‘Essence Purple’ and *Helichrysum italicum* (Roth) G.Don was provided by the company “Cappello Roberto” located in Albenga (Savona, Liguria Region, Italy) in November 2024. Every year, this company cultivates approximately 140,000 pots of *Lavandula* (Figure 12a), which are pruned two or three times between September and March, and about 30,000 pots of *Helichrysum* (Figure 12b), which are pruned once or twice during the same period. Due to different growth rates of the plants, the lavender was pruned during the flowering stage, whereas the helichrysum was pruned during the vegetative stage. Pruning is carried out mechanically in order to trim only the apical parts and give the plant a rounded and compact shape suitable for commercial distribution (Figure 12c). The pruning waste (Figure 12d) was taken to the laboratory in paper bags and subsequently air-dried at room temperature before being subjected to hydrodistillation for EO extraction.

### 4.2. Micromorphological and Anatomical Analyses

Small fresh portions of the corolla surface of *Lavandula angustifolia* and hand-made transverse sections of fresh leaves of *L. angustifolia* and *Helichrysum italicum*, obtained using a double-edged razor blade, were observed either mounted in water or stained with the metachromatic dye Toluidine Blue O, pH 4.4 [71,72] and Fluorol Yellow 088 [73]. Some hand-made transverse sections of the leaves of *H. italicum* were cleared with an aqueous solution of chloral hydrate and mounted in a chloral hydrate–glycerol mounting medium to prevent crystallization of the reagent [74]. All samples were observed using a Leica DM 2000 fluorescence microscope equipped with an H3 filter (excitation filter BP 420–490 nm) (Leica Microsystems, Wetzlar, Germany) and a ToupCam digital camera (CMOS sensor, 3.1 MP resolution ToupTek Photonics, Hangzhou, China).

Moreover, small samples of the aerial portion of both species were fixed in FineFIX working solution (Milestone SRL, Sorisole, Bergamo, Italy), left overnight at 4 °C, dehydrated in a graded ethanol series (70, 80, 90, and 100%) for 1 h each [75], and finally critical-point dried using liquid carbon dioxide (CO_2_) (K850CPD 2M, Strumenti S.r.l., Roma, Italy). The dried specimens were mounted on aluminum stubs using double-sided adhesive carbon tape and coated with a 10 nm layer of gold [76]. The examination was performed using a VEGA3 Tescan type LMU Scanning Electron Microscope (Tescan USA Inc., Cranberry Twp, PA, USA), operating at an accelerating voltage of 20 kV.

### 4.3. Essential Oils Extraction

Pruning material from both plants was subjected to steam distillation for 2 h, following the procedure reported in the European Pharmacopoeia [77]. The EOs obtained were subsequently dissolved in *n*-hexane, filtered through anhydrous sodium sulphate, and evaporated under a gentle nitrogen (N_2_) stream to remove the residual solvent. The purified EOs were stored in amber glass vials at +4 °C, protected from light, heat, and moisture, until further analysis.

### 4.4. Gas Chromatography with Flame Ionization Detection (GC-FID) and Gas Chromatography–Mass Spectrometry (GC-MS) Analyses

The composition of EOs was studied by GC-FID and GC-MS analysis. For GC-FID analysis, a Perkin-Elmer Sigma 115 gas chromatograph (Waltham, MA, USA) equipped with a non-polar HP-5MS fused silica capillary column (30 m × 0.25 mm i.d.; 0.25 μm film thickness) was used. For GC–MS analysis, an Agilent 6850 Series II gas chromatograph (Agilent, Santa Clara, CA, USA) coupled with an Agilent 5973 mass selective detector was employed, using an HP-5MS fused silica capillary column (Agilent, 30 m × 0.25 mm i.d.; 0.25 μm film thickness). The mass spectrometer was operated with electron impact (EI) ionization at 70 EV, and an ion multiplier voltage was set at 2000 V. Mass spectra were acquired over a mass range of 40–500 amu at a rate of five scans per second. GC-FID and GC-MS analyses were performed under the same chromatographic conditions. The injector temperature was 250 °C; the FID detector temperature was 290 °C, whereas the MS quadrupole temperature was set at 150 °C. The oven temperature program was set as follows: initial isothermal phase at 40 °C for 5 min, followed by an increase at 2 °C/min up to 270 °C, with a final isothermal hold at 270 °C for 20 min. To support compound identification, GC-FID and GC-MS analyses were also performed on an HP Innowax polar column (50 m × 0.20 mm i.d.; 0.25 μm film thickness) under the same chromatographic conditions. Helium was used as the carrier gas in the analyses at a constant flow rate of 1.0 mL/min. Phytochemicals were identified by comparing their Kovats retention indices (KI) with those reported in the literature [78,79,80,81] and by careful comparison of the mass spectra with those in the NIST 17 and Wiley 257 mass spectral libraries [82]. Kovats indices were determined relative to a homologous series of *n*-alkanes (C10–C35), analyzed under the same operating conditions. Relative concentrations of the components were calculated by peak area normalization, without the use of response correction factors.

### 4.5. Phytotoxic Activity

Phytotoxic effects were assessed by evaluating seed germination and radical elongation of two crop species, *Raphanus sativus* L. (radish) and *Hordeum vulgare* L. (barley), and two weeds, *Lolium multiflorum* Lam. (Italian ryegrass) and *Sinapis alba* L. (wild mustard). Seeds of *R. sativus* and *H. vulgare* were purchased from Blumen Group S.r.l. (Bologna, Italy), while seeds of *L. multiflorum* were obtained from Fratelli Ingegnoli S.p.a. (Milan, Italy). Seeds of *S. alba* were collected from natural wild populations. These species are commonly used in phytotoxicity assessments due to their rapid germination rate and well-characterized physiological responses. Before testing, seeds were sterilized with 95% ethanol for 15 s and then placed in Petri dishes (Ø 90 mm) containing three layers of Whatman filter paper, soaked with 7 mL of deionized water (control) or 7 mL of EO solution at various concentrations. The germination tests were carried out at 20 ± 1 °C under natural photoperiod conditions. To improve solubility, the EO was dissolved in a water–acetone mixture (99.5:0.5, *v*/*v*) and tested at concentrations of 500, 250, 125 and 63 μg/mL. Seeds were preliminarily tested in deionized water to verify their germination capacity. Seed germination was monitored at 24 h intervals, and a seed was considered germinated when radicle protrusion was visible [83]. After 120 h for *R. sativus*, *S. alba*, and *H. vulgare* and 168 h for *L. multiflorum*, germination percentage was recorded and radicle length was measured (cm). Each treatment was performed in triplicate using Petri dishes containing 10 seeds each. Control assays containing the solvent mixture (water–acetone, 99.5:0.5 *v*/*v*) were also performed to exclude solvent effects.

### 4.6. Ecotoxicological Assays

To obtain a preliminary evaluation of the environmental safety of the investigated EOs, two ecotoxicological assays based on aquatic invertebrates were performed using *Artemia salina* and *Daphnia magna* as model organisms.

The results were expressed as percentage viability for *A. salina* and percentage immobilization for *D. magna* (mean ± standard deviation, SD). All experiments were independently performed three times, with each concentration tested in triplicate for the *A*. *salina* assay and in quadruplicate for the *D. magna* assay.

#### 4.6.1. *Artemia salina* Lethality Assay

The general toxicity of the EOs was assessed using the *Artemia salina* lethality bioassay according to a standardized protocol [84]. *Artemia* cysts were hatched in artificial seawater (3% salinity; 33.33 g sea salt per L of tap water) under continuous aeration and illumination at room temperature for 36–48 h. Newly hatched nauplii were collected and used for the bioassay. Stock solutions of the EOs were prepared in DMSO and serially diluted to obtain final concentrations of 1, 0.5, 0.25, 0.125, 0.0625, and 0.03125 mg/mL in seawater, with a final DMSO concentration of 0.1%.

For the assay, 10 nauplii were transferred into each well of a 24-well plate containing 2 mL of artificial seawater. Test solutions were added to the wells, while DMSO (0.1%) served as the negative control, and potassium dichromate (K_2_Cr_2_O_7_, 50 µg/mL final concentration) was used as positive control. The plates were incubated for 48 h at room temperature under continuous illumination and gentle aeration. Mortality was recorded after 24 and 48 h of exposure by observing the larvae under a stereomicroscope. Nauplii that showed no movement after gentle agitation were considered dead.

#### 4.6.2. *Daphnia magna* Acute Toxicity Test

The acute toxicity of the EOs toward freshwater invertebrates was evaluated using neonates of *Daphnia magna* by means of the commercial DAPHTOXKIT F magna test kit (Ecotox LDS, Cornaredo, Milano, Italy). Dormant eggs (ephippia) of *D. magna* were hatched according to the manufacturer’s instructions in standard freshwater medium. The medium was obtained by dissolving NaHCO_3_, CaCl_2_·2H_2_O, MgSO_4_·7H_2_O, and KCl in deionized water. Ephippia were incubated in Petri dishes containing pre-aerated freshwater for 72 h at 20–22 °C under continuous illumination (≥6000 lux provided by an illuminated light table used for microbiotests) to obtain neonates suitable for testing. Only neonates less than 24 h old were used for the toxicity assays. Stock solutions of the EOs were prepared by dissolving them in DMSO and then diluted in standard freshwater to obtain the following concentrations: 1, 0.5, 0.25, 0.125, 0.0625, and 0.03125 mg/mL.

The assay was performed in multi-well plates supplied with the kit. For each concentration and control, four replicates were prepared, each containing 5 neonates in 10 mL of test solution. Control wells contained standard freshwater with the same solvent concentration used in the test solutions (0.1% DMSO), while 3 μg/mL K_2_Cr_2_O_7_ was used as positive control. The plates were sealed and incubated at 20 °C in the dark. Immobilization was evaluated after 24 and 48 h using a stereomicroscope. Organisms that were unable to swim within 15 s after gentle agitation were considered immobilized. The percentage of immobilization was calculated for each concentration, and the test was considered valid when immobilization in the control did not exceed 10%.

### 4.7. Statistical Analysis

Before performing parametric analyses, data normality was assessed using the Shapiro–Wilk test. Differences among treatments in both phytotoxicity and ecotoxicological assays were analyzed by one-way analysis of variance (ANOVA) using GraphPad Prism 6.0 (GraphPad Software Inc., San Diego, CA, USA). The results were compared with the negative control and were considered statistically significant at *p* < 0.05 when using Dunnett’s multiple comparison test.

For the ecotoxicological assays, median effective concentrations (EC_50_) and the corresponding 95% confidence intervals were calculated from concentration–response curves using a four-parameter logistic non-linear regression model implemented in GraphPad Prism (version 10.0; GraphPad Software, Boston, MA, USA).

## 5. Conclusions

The present study demonstrates that pruning residues derived from ornamental cultivations of *Lavandula angustifolia* Mill. ‘Essence Purple’ and *Helichrysum italicum* (Roth) G.Don represent a valuable secondary biomass that can be exploited for the recovery of bioactive EOs. Micromorphological investigations confirmed the persistence of abundant glandular trichomes in the aerial portions of the pruning material, indicating that the structures responsible for the biosynthesis and accumulation of volatile metabolites remain well preserved, even in plant residues. The phytochemical characterization revealed that the chemical profiles of the EOs obtained from pruning biomass are generally consistent with those reported for the respective species, although some quantitative variations were observed, likely related to the heterogeneous nature of the plant material and to the physiological stage of the plants at the time of pruning.

Notably, the observed compositional differences, particularly the relatively higher contribution of sesquiterpenes, further support the concept that pruning-derived biomass may provide chemically differentiated EOs compared to those obtained from conventional plant organs.

The biological assays highlighted the potential biological relevance of these volatile fractions. The phytotoxic tests showed that the essential oils were able to influence early plant development processes, particularly radicle elongation, suggesting that the relatively high proportion of sesquiterpene compounds may play a relevant role in the observed activity. However, the concentrations tested should be interpreted as indicative of intrinsic phytotoxic potential under controlled conditions, and further studies are required to assess their effective applicability under agronomic scenarios.

In parallel, the preliminary ecotoxicological evaluation performed on aquatic invertebrates revealed a limited effect on *Artemia salina* and a higher sensitivity in *Daphnia magna*, highlighting species-specific responses and suggesting that membrane-mediated mechanisms may contribute to the observed effects. This provides useful information on the biological activity of these natural mixtures and on the concentration ranges potentially compatible with agronomic applications, although a more comprehensive environmental risk assessment will be necessary before any practical use can be envisaged.

Overall, these findings support the concept that pruning residues from the cultivation of medicinal and aromatic plants should not be considered merely as horticultural waste, but rather as a promising source of biologically active natural products. The present study should be considered as a preliminary proof-of-concept within a broader research framework aimed at evaluating the phytotoxic potential of EOs and plant extracts obtained from pruning residues of aromatic plants. Future investigations will include the analysis of additional EOs and extracts, and only the most promising phytocomplexes will be subsequently evaluated under field conditions to assess their potential application as natural bioherbicides in sustainable agricultural systems.

## Figures and Tables

**Table 1:** Chemical composition of the essential oil from *Lavandula angustifolia* Mill. ‘Essence Purple’ determined by GC-FID and GC–MS analysis.

N.	Compound	%	Ki ^a^	KI ^b^	Identification ^c^
**1**	α-Pinene	0.77	933	1036	1, 2, 3
**2**	Camphene	0.34	945	1083	1, 2, 3
**3**	β-Pinene	3.58	973	1120	1, 2, 3
**4**	1-Octen-3-ol	0.24	983	1452	1, 2
**5**	β-Myrcene	0.54	992	1145	1, 2, 3
**6**	α-Phellandrene	0.28	999	1165	1, 2, 3
**7**	3-Carene	3.01	1005	1171	1, 2
**8**	*m*-Cymene	0.48	1018	1254	1, 2
**9**	*p*-Cymene	0.88	1021	1234	1, 2, 3
**10**	Limonene	3.67	1024	1180	1, 2, 3
**11**	*trans*-β-Ocimene	1.48	1038	1242	1, 2, 3
**12**	Linalool	7.37	1099	1506	1, 2, 3
**13**	*trans*-Pinocarveol	0.44	1132	1664	1, 2
**14**	Camphor	0.38	1136	1491	1, 2, 3
**15**	Pinocarvone	0.25	1156	1586	1, 2
**16**	*p*-Mentha-1,5-dien-8-ol	0.31	1159	1670	1, 2
**17**	*endo*-Borneol	2.81	1162	1715	1, 2, 3
**18**	Lavandulol	0.50	1168	1686	1, 2
**19**	Terpinen-4-ol	0.41	1174	1636	1, 2, 3
**20**	Cryptone	1.13	1182	1659	1, 2
**21**	α-Terpineol	0.27	1190	1662	1, 2, 3
**22**	Myrtenal	0.39	1192	1648	1, 2
**23**	Myrtenol	0.34	1195	1804	1, 2
**24**	Eucarvone	0.54	1200		1, 2
**25**	4-(1-Methylethyl)-benzaldehyde	0.68	1234	1753	1, 2
**26**	Linalyl acetate	14.07	1260	1542	1, 2
**27**	Phellandral	0.25	1271	1720	1, 2
**28**	Lavandulol acetate	2.56	1295		1, 2
**29**	Thymol	0.25	1300	2172	1, 2, 3
**30**	Carvacrol	0.51	1303	2219	1, 2, 3
**31**	Nerol acetate	0.41	1367	1731	1, 2
**32**	*cis*-Geranyl acetate	2.37	1388	1746	1, 2
**33**	α-Cedrene	0.45	1400		1, 2
**34**	Caryophyllene	1.47	1410	1617	1, 2
**35**	α-Santalene	1.32	1414	1597	1, 2
**36**	α-Bergamotene	0.32	1431		1, 2
**37**	β-Copaene	0.41	1440		1, 2
**38**	β-Cedrene	1.47	1457	1573	1, 2
**39**	Germacrene D	0.38	1476	1712	1, 2
**40**	Helminthogermacrene	0.70	1500		1, 2
**41**	γ-Cadinene	12.07	1510	1752	1, 2
**42**	Cadina-1,3,5-triene	0.87	1516		1, 2
**43**	Cadala-1(10),3,8-triene	0.24	1537		1, 2
**44**	Caryophyllene oxide	0.98	1577	2000	1, 2
**45**	α-*epi*-7-*epi*-5-Eudesmol	0.26	1597		1, 2
**46**	Epicubenol	2.00	1609	2025	1, 2
**47**	τ-Cadinol	23.09	1642	2187	1, 2
**48**	8a-Isopropyl-3-methyl-1,2,4,5,8,8a-hexahydroazulene-6-carbaldehyde	0.25	1655		1, 2
**49**	6-Isopropenyl-4,8a-dimethyl-1,2,3,5,6,7,8,8a-octahydro-naphthalen-2-ol	0.45	1660		1, 2
**50**	Muurol-5-en-4-one <*cis*-14-*nor*->	0.89	1682		1, 2
**51**	Ylangenal	0.55	1738		1, 2
	Total	99.68			
	Monoterpene hydrocarbons	15.03			
	Oxygenated monoterpenes	35.36			
	Sesquiterpene hydrocarbons	19.70			
	Oxygenated sesquiterpenes	28.47			
	Others	0.92			

^a, b^ The Kovats retention indices are relative to a series of *n*-alkanes (C10–C35) on the non-polar HP-5MS and the polar HP Innowax capillary columns, respectively. ^c^ Identification method: 1 = comparison of Kovats retention indices with published data; 2 = comparison of mass spectra with those reported in the NIST 17 and Wiley 275 libraries, and with published data; 3 = co-injection with authentic standards.

**Table 2:** Chemical composition of the essential oil from *Helichrysum italicum* (Roth) G.Don determined by GC-FID and GC–MS analyses.

N.	Compound	%	Ki ^a^	Ki ^b^	Identification ^c^
**1**	α-Pinene	3.65	934	1036	1, 2, 3
**2**	Limonene	0.19	1024	1180	1, 2, 3
**3**	Linalool	0.29	1097	1506	1, 2, 3
**4**	(Z)-2-Methylbutyl 2-methylbut-2-enoate	0.16	1154	1469	1, 2
**5**	4,6-Dimethyloctane-3,5-dione	0.39	1188	1597	1, 2
**6**	α-Terpineol	0.22	1189	1662	1, 2, 3
**7**	Nerol	0.90	1227	1781	1, 2
**8**	4-Methyl-amyl angelate	1.04	1289	1471	1, 2
**9**	Thymol	0.29	1295	2172	1, 2, 3
**10**	Carvacrol	0.51	1305	2219	1, 2, 3
**11**	Eugenol	0.30	1357	2186	1, 2, 3
**12**	Neryl acetate	8.33	1370	1746	1, 2
**13**	Isoitalicene	1.40	1371		1, 2
**14**	Italicene	8.42	1396	1536	1, 2
**15**	β-Caryophyllene	4.42	1410	1617	1, 2
**16**	2E-Nonenyl angelate	0.22	1422		1, 2
**17**	*trans*-α-Bergamotene	1.89	1432	1536	1, 2
**18**	α-Humulene	0.35	1447	1641	1, 2
**19**	Neryl propionate	3.48	1457	1764	1, 2
**20**	4-*epi*-α-Acoradiene	0.96	1462		1, 2
**21**	β-Chamigrene	0.19	1471	1701	1, 2
**22**	γ-Curcumene	15.47	1483	1664	1, 2
**23**	α-Curcumene	3.15	1485	1786	1, 2
**24**	α-Selinene	0.28	1492	1688	1, 2
**25**	α-Zingiberene	0.16	1496	1745	1, 2
**26**	β-Bisabolene	0.43	1505	1741	1, 2
**27**	β-Curcumene	0.85	1508		1, 2
**28**	Cadina-3,9-diene	0.76	1512	1720	1, 2
**29**	Selina-3,7(11)-diene	2.08	1528	1796	1, 2
**30**	β-Maaliene	0.27	1533	1671	1, 2
**31**	2-Phenylethyl tiglate	0.21	1537	2226	1, 2
**32**	*trans*-α-Bisabolene	0.24	1541		1, 2
**33**	Germacrene B	0.17	1550	1805	1, 2
**34**	*trans*-Nerolidol	0.16	1565	2008	1, 2
**35**	Caryophyllene oxide	0.67	1577	1983	1, 2
**36**	Neryl isovalerianate	0.51	1589	1872	1, 2
**37**	10,10-Dimethyl-2,6-dimethylenebicyclo [7.2.0] undecane	1.20	1594		1, 2
**38**	Guaiol	5.83	1595	2094	1, 2
**39**	Rosifoliol	7.37	1603	2133	1, 2
**40**	Agarospirol	0.66	1624		1, 2
**41**	Hinesol	0.24	1635	2228	1, 2
**42**	Eudesm-4(14)-en-11-ol	10.71	1647	2230	1, 2
**43**	γ-Eudesmol	3.11	1650	2182	1, 2
**44**	α-Eudesmol	2.05	1655		1, 2
**45**	Palustrol	0.23	1659	1938	1, 2
**46**	γ-Himachalene	0.17	1661	1737	1, 2
**47**	Guai-1(10)-en-11-ol	1.85	1665	2265	1, 2
**48**	β-Bisabolol	0.62	1670		1, 2
**49**	α-Bisabolol	0.24	1685	2232	1, 2
**50**	Z-α-*trans*-Bergamotol	1.09	1688	2242	1, 2
**51**	Eudesm-7(11)-en-4-ol	0.21	1692	2241	1, 2
**52**	(Z)-3,7-Dimethylocta-2,6-dien-1-yl hexanoate	0.29	1699	2033	1, 2
**53**	3-(1,5-Dimethylhex-4-en-1-yl)-2,2-dimethylcyclopent-3-en-1-ol	0.17	1735		1, 2
**54**	2,6-Dimethylocta-2,6-diene-1,8-diyl diacetate	0.30	1740		1, 2
**55**	(Z)-7-Hexadecenal	0.22	1783	2144	1, 2
	Total	99.57			
	Monoterpene hydrocarbons	3.84			
	Oxygenated monoterpenes	11.3			
	Sesquiterpene hydrocarbons	42.86			
	Oxygenated sesquiterpenes	35.04			
	Others	6.53			

^a, b^ The Kovats retention indices are relative to a series of n-alkanes (C10–C35) on the non-polar HP-5MS and the polar HP Innowax capillary columns, respectively. ^c^ Identification method: 1 = comparison of Kovats retention indices with published data; 2 = comparison of mass spectra with those reported in the NIST 17 and Wiley 275 libraries, and with published data; 3 = co-injection with authentic standards.

**Table 3:** Percentage of phytotoxic activity of *Lavandula angustifolia* Mill. ‘Essence Purple’ EO. Heat map representation of inhibition values relative to the control treatment. Green indicates positive inhibition values, red indicates negative values (stimulatory effect), and white indicates the control or absence of activity (0.0%). Color intensity reflects the magnitude of the effect.

Number of Germinated Seeds				
	*Hordeum vulgare*	*Raphanus sativus*	*Lolium multiflorum*	*Sinapis alba*
Control H_2_O + C_3_H_6_O	0.0	0.0	0.0	0.0
Treatment (µg/mL)				
63	5.4	7.0	−24.3	−16.2
125	−4.3	0.0	0.0	−3.8
250	14.0	7.0	10.0	66.2
500	38.7	17.0	4.3	100
**Radicle Length (cm)**				
	*Hordeum vulgare*	*Raphanus sativus*	*Lolium multiflorum*	*Sinapis alba*
Control H_2_O + C_3_H_6_O	0.0	0.0	0.0	0.0
Treatment (µg/mL)				
63	9.1	−15.8	−105.6	−62.5
125	−72.7	−18.4	−27.8	−25.0
250	−140.9	−23.7	16.7	62.5
500	−122.7	47.4	50.0	100

**Table 4:** Percentage of phytotoxic activity of *Helichrysum italicum* (Roth) G.Don EO. Green indicates positive inhibition values, whereas red indicates negative inhibition values (i.e., stimulation rather than inhibition). White indicates the control treatment and the absence of activity (0.0%). Color intensity is proportional to the magnitude of the observed effect.

Number of Germinated Seeds				
	*Hordeum vulgare*	*Raphanus sativus*	*Lolium multiflorum*	*Sinapis alba*
Control H_2_O + C_3_H_6_O	0.0	0.0	0.0	0.0
Treatment (µg/mL)				
63	3.2	0.0	−4.3	−3.8
125	17.2	0.0	−32.8	−12.5
250	24.7	7.0	−10.0	0.0
500	24.7	3.0	−18.6	3.8
**Radicle Length (cm)**				
	*Hordeum vulgare*	*Raphanus sativus*	*Lolium multiflorum*	*Sinapis alba*
Control H_2_O + C_3_H_6_O	0.0	0.0	0.0	0.0
Treatment (µg/mL)				
63	−113.6	−26.3	−94.4	−37.5
125	−131.8	−31.6	−122.2	−62.5
250	−118.1	2.6	−27.8	0.0
500	−22.7	26.3	27.8	25.0

## Data Availability

The original contributions presented in this study are included in the article. Further inquiries can be directed to the corresponding author.

## Abbreviations

The following abbreviations are used in this manuscript:

EO	Essential oil
MAPs	Medicinal and aromatic plants
NGTs	Non-glandular trichomes
GTs	Glandular trichomes
PGTs	Peltate glandular trichomes
CGTs	Capitate glandular trichomes
BGTs	Biseriate glandular trichomes
GC–FID	Gas chromatography–flame ionization detection
GC–MS	Gas chromatography–mass spectrometry
KI	Kovats retention index
DMSO	Dimethyl sulfoxide
SD	Standard deviation
ANOVA	Analysis of variance
EOs	Essential oils
EC_50_	Median effective concentration
